# Supplementary material for: “Just a knife wound this week, nothing too painful”: An ethnographic exploration of how primary care patients experiencing homelessness view their own health and healthcare
Source: PLoS One. 2024 Jul 9;19(7):e0299761. doi: 10.1371/journal.pone.0299761 (PMC11232971; doi:10.1371/journal.pone.0299761)
Supplement: S1 Table — (DOCX) [file pone.0299761.s002.docx]

| **Theme I. Self-Identified Priorities** | | |
| --- | --- | --- |
| **Sub-Theme** | **Code** | **Sample Excerpts** |
| Mental Health | Mental disorders | Eimear says it’s hard for her to relate to the women she is around due to the severity of her injuries and experiences. She asks me at one point, “What’s wrong with me?” She says she’s having nightmares all the time, that she feels panicked and anxious. She says she wants peace, that she’s ready to stop trying, that she thinks her kids’ lives might be simpler without her. (**Eimear, In-Depth Conversation**)  Tristan is hearing voices. “They’re telling me to do bad things.” He doesn’t want to see a mental health nurse, saying “I’ll be alright.” (**Tristan, In-Depth Conversation**) |
|  | Dependency on psychotropics | Kieron says he’s pretty happy with the medications he’s on at the minute. He has severe anxiety and doesn’t know how he’d function without his anti-anxiety meds. He needs them to be able to leave the house, to be in crowds. “Without me tablets I’m hopeless.” (**Kieron, In-Depth Conversation**) |
|  | Competing emotions | “Well, I’m really having a hard time. I lost me sister recently and the hostel I was staying at, where I had friends, kicked me out on Friday and I don’t know why. They wouldn’t tell me why. And the new hostel I’m in, it’s not as good. I’m sharing a room with a girl I don’t know. And my phone broke, so I can’t get in touch with anyone.” She explains she feels panicky [she is in the clinic today to get medication] all the time and that her nerves are really bad. Through a lot of this conversation, she is crying. She asks me for a tissue. (**Pamela, In-Depth Conversation**) |
|  | Communication challenges | Kevin is visibly drowsy. There is drool coming from his mouth and he seems uninterested in surroundings, with his eyes glazed over. When members of staff, or those around him, try to speak to him he manages no more than a grunt in reply. At the end of my visit, I learn from staff that he is on antipsychotic medication which is affecting his speech. (**Observation**) |
|  | Loss and grief | Sean suffered from crack cocaine addiction for 30 years but made a complete recovery. In the past year, he turned to alcohol (and eventually alcoholism) in relation to his dog being taken away and his sister committing suicide around the same time. He’s in the clinic for Librium to help with the anxiousness he has been feeling lately. (**Sean, In-Depth Conversation**)  Woman 3 is speaking to a friend. She said goodnight to her roommate the other night and woke up to find her dead in the hostel room. “I’m the one who found her. I went over to wake her up and saw one side of her face was black. All night I slept with her like that” (**Observation**). |
| Relationships | Parenting | She has a son who has lived with her in the past but does not live with her now. Nora says, “If there is one gap in services, it is social workers for single mothers recovering from addiction whose children are returned to them.” She understands that the priority has to be the child but feels there needs to be a better understanding of the mom’s addiction as well. As she’s leaving, we greet each other. She tells me to let a member of staff know that he has to check her urine for her medication. She’s worried that if anything is misreported, she won’t get to see her son. (**Nora, In-Depth Conversation**) |
|  | Family Ties | Ryan is concerned that his family are back to not talking to him again. His nephew’s christening is soon but Ryan isn’t invited. He says he’ll drop off a gift anyway. He says he was using the family’s [television] account but that someone changed the password. “Isn’t that petty”, he says, “The whole family’s using it, but they have to change it because of me.” (**Ryan, In-Depth Conversation**) |
|  | Intimate Relationships | Cathleen is incredibly upset when explaining that her partner punched her last night. She says that it’s the crack. “He would never have done that before. That’s not him.” (**Cathleen, In-Depth Conversation**)  A woman tells her friends that ‘her man’ is getting out next week, saying ‘you know what that means.’ She feels nervous about this. (**Observation**) |
| Women’s Safety | Domestic, Sexual, and Gender-Based Violence | As we’re speaking, she starts to break down. She tells me that the man who violently assaulted her just got out of prison on compassionate grounds. “Where is the compassion for me?!” she says. She saw him on the street that morning and had to get off the bus to get sick. (**Eimear, In-Depth Conversation**)  Cara starts by saying that more attention needs to be paid to domestic violence/sexual assault. She had been assaulted by a partner and eventually went to a women’s shelter. Only, her attacker found her there and the shelter threw her out immediately. She then became homeless, living out of her car for a while. She says that being homeless is very rough for a woman. “If you have drugs in your bra or your panties, those guys are not afraid to come and find it there.” The police ended up taking her car and she slept on the floor of a harm reduction NGO for a while. I ask her how she stayed safe from her attacker when she was thrown out of the shelter. She looks at me, kind of smirks, and pops out her dental implant, revealing a missing front tooth. (**Cara, In-Depth Conversation**) |
| Housing and Income | Accessing satisfactory short-term accommodation | Jamie – after being on hold for 40 minutes - is told on the Free phone that he can’t access a bed for the night because he’s not from Dublin. He’s advised to go home to his home county (which he will not do). He’s sleeping rough but without a tent because he doesn’t have a place to store one during the day. I notice that his duffle bag is heavy and hard to carry around all day while he is on crutches. (**Observation**) |
|  | Accessing long-term accommodation | He speaks to me for a while, lots about how he got his injured foot (fighting I think?) and – when I tell him I do research – he says that homelessness is an enormous issue. He has a place to live now but didn’t for a long time and says the streets are a tough place. He says that crack is everywhere. (**Luke, Casual Conversation**)  He says he’s trying to get an apartment through HAP [Housing Assistance Payment] but was told that he didn’t fit the demographic. He is very far down the list to getting his own apartment. (**Sean, In-Depth Conversation**) |
|  | Lack of meaningful activities | Sean laughs a lot, despite everything. He says he’s bored. Bored in the clinic and bored in his life. His mobility has been reduced since his injury. He tells me that he feels affected by the Librium. He says he’s glad I am there to help the time pass as he waits anyway. (**Sean, In-Depth Conversation**) |
|  | Social stigma | Sean tells me that he tried to ring social services this week and was hung up on. He shows me a card with a list of numbers to call – at least 10 – it’s very confusing. He says when he finally got someone on the phone he said, six times, “Please don’t hang up on me”, but the person did. He tried to set up an account this week at the Credit Union. The girl behind the counter kept asking him questions that didn’t make sense for someone in homelessness. He says there’s a lot of people in those type of positions that don’t know what to make of him. (**Sean, In-Depth Conversation**) |
| Physical Health | Pain (Wounds, Withdrawal) | Gavin is in for, "Just a knife wound this week, nothing too painful." He explains that he won't go to the hospital anymore because he'll have to give up the name of the guy who stabbed him. When he's back out on the street, that guy will send somebody for him. "No chance I'm doing that." He shows me how he took care of things himself with superglue. (**Gavin, Casual Conversation**)  Wyatt comes in with his fiancé. He does all the talking. His legs are infected, he’s wrapped them up with cellotape and toilet paper and shows them multiple times. He says he is in a lot of pain. (**Observation**)  Man 12, who I've seen several times in the clinic, is going through withdrawal as he sits. He shows a friend that his hands are shaking and I see visible tremors in his face. After a few minutes, he leaves with a "fuck this." When he comes back about 30 minutes later, he seems better, calmer. He says he found €2 on his way out the door which was a "gift from God." With it, he was able to buy something to drink (I learn later from a member of staff that the cheapest option is a bottle of some type of floor cleaner with a high alcohol content). (**Observation**) |
|  | Sleep | This morning, three different people mention sleeplessness or being exhausted in the waiting room (**Observation**).  Eimear has been having nightmares and trouble sleeping. (**Eimear, In-Depth Conversation**) |
|  | Disability | Sean is in his late 60s and has a broken hip for which he uses a crutch. He has broken his arm/wrist multiple times, making it uncomfortable to use the crutch for his hip. (**Sean, In-Depth Conversation**)  She tells me that she was dyslexic and had a hard time in school growing up. (**Aileen, In-Depth Conversation**)  Eimear went into a coma at some point. She says her brain function hasn’t been the same since. She said she died several times. She apologises for getting mixed up in her timeline, saying "I can’t focus, or communicate things in order anymore." (**Eimear, In-Depth Conversation**) |
|  | Other health concerns | Tyler says he has HIV, diagnosed [for many years]. He takes two tablets a day. “Don’t forget that undetectable means untransmissible!” He says the tablets allowed his immune system to improve enough to be able to fight off Hep C as well, so he is currently Hep C free. He says the stigma against HIV is getting a little better over time. (**Tyler, Casual Conversation**)  Man 15 mentions needing to see a dentist to a member of staff but was told he couldn't access the free, low threshold service because he has a medical card. (**Observation**) |
|  | Rapid decline | Tierney is back this week with a key worker. He’s not doing well at all. He seems very frail and is sitting on his stretcher, moaning. He has wet himself. The nurse gives him something to drink and makes sure he finishes his breakfast. He says, ‘I don’t feel well’ many times. It’s very hard to see the difference in him since the last time I chatted with him. [Later excerpt] I learn that Tierney fatally overdosed…. Allegedly, it was another client who dealt him the drugs. (**Tierney, Observation**) |
| Criminal Justice | Court dates | He says his biggest concern is regarding his upcoming court dates and how he can take care of his kids if he goes to prison. (**Kieron, In-Depth Conversation**) |
|  | Prison | He is set to go into rehab for the first time in January and has over ten court dates lined up in the meantime. If the judges find him guilty, he will go to prison and not to rehab. Leonard has been to prison before and says it’s not too bad there. He can work out, get free meals and a travel ticket upon release, and all of his friends are there. “I can go see the boys!” he jokes. He is not unhappy about the possibility of going to prison. As he says, "It's fair really. I've caused a lot of trouble from the drinking." (**Leonard, In-Depth Conversation**) |
|  | Stigmatising interactions | In the past year, Sean was sleeping rough and was awoken by the guards for being “drunk and disorderly”, which he doesn’t agree that he was, being asleep after all. Sean had a dog for years who was “his baby”. When the guards woke him, they took her. They also cut off his pants, which contained his wallet and ID, and never returned these to him. Sean believes someone reported him despite how carefully he looked after his dog, “She was in better shape than me I tell ya, gave her the food off me own plate.” He doesn’t understand why someone would make false claims against him. (**Sean, In-Depth Conversation**)  A member of staff asks if she’s gone to the guards after running into the man who assaulted her. “What for?” she says, “They’ll just say I’m some junkie.” (**Eimear, Observation**) |
| Navigating Addiction | Isolation while in addiction | I ask Jack if there’s anything he wishes more people understood about his life. He thinks for a second, “Oh you know, people walk right past you and don’t see you. I counted once… 90 people and no one gave a thing. I made a bet with a few of the girls standing around and was right. 8 out of 10 people will ignore you completely; 1 will look you in the eyes and say sincerely that they’re sorry but don’t have it; and 1 might, might give you something.” (**Jack, In-Depth Conversation**) |
|  | Breaking point | “I’ve been in Dublin for years and I hate it. I think it’s disgusting. There are drugs everywhere and this addiction I have is a borne on top of me and I can’t get rid of it.” She says she doesn’t see the point of any of this anymore and doesn’t know how to change or what to do. “There's just no way I'm getting through the next few days sober.” She looks out the window and points to the dealers across the street. She says they’re everywhere, that you can’t walk down the street and not see it. As well, she says the girl in the hostel is using crack in the room. Towards the end of our conversation, Pamela gets up abruptly. “I’m really sorry but I need to go out and have a cigarette. I can’t sit still for too long.” (**Pamela, In-Depth Conversation**) |
|  | Accessing treatment | A man who comes regularly to the clinic is working to reduce his methadone dose so as to enter residential treatment. One visit, he comes in very upset. He tells a member of staff that he was scheduled to enter residential treatment that Friday but just learned that he would have to get through the weekend to go in the following Monday. This seemed to be the straw that broke the camel's back. I overhear him say, "If I'm going to use, today's going to be the day." Clinic staff speak with him, working hard to help him calm down. (**Observation**) |
| **Theme II. Satisfaction with Services** | | |
| **Sub-Theme** | **Codes** | **Sample Excerpts** |
| Primary care and addiction service | Social setting | Woman 13 is in accompanying Eimear and Teresa warns her against a particular stabilisation facility. The woman takes her advice. She was going to go in soon, but now won’t after being told, ‘That place is a kip.’ (**Observation**)  Aine doesn't need to see the GP but has come in to use her friend's phone. (**Observation**) |
|  | Relationships with staff/providers | I talk to Róisín. She says she likes the clinic a lot, that she can finally be honest. She said that before, with GPs, she was never honest for fear of being judged. She says someone at a homeless service told them about the clinic four months ago and they’ve been coming ever since. She says she can be honest now and – as a result – is feeling better. She started methadone four months ago and that has been going well as well…She says, “It feels really good to be trusted. It feels good that people here know my name.” (**Róisín, In-Depth Conversation**) |
|  | Wait times | I’m seated next to Paul who is talking to Tierney, also in his 50s-60s who uses a walker (also his chair). The two are catching up with each other. They talk about having scrambled eggs for breakfast. They become agitated by the wait time (about 30 minutes). Paul says, “I’d rather run down for a bet and a pint.” He seems pleased that his local is now open earlier in the day. He stays but after ten more minutes, Tierney gives up waiting and leaves. (**Observation**) |
|  | Chaotic environment | Michael comes in. I see him nearly every Monday I’m in. Generally, he is quite nice. He does like to ‘pretend wrestle’ with other clients. Today, he’s extremely agitated. His hands are shaking, he’s speaking very loudly, and there is foam at his mouth. He says he needs to see the GP immediately. He’s nearly yelling. A member of staff tells him to sit down. He comes to the chair next to me, at this point he’s shaking intensely. He says no one would give him weed which is why he’s panicking. He needs something. He takes off his sweatshirt and I see scars all up and down his arms, deep scars. On his right arm is a bandage, now covered in blood. He says to look at the bandage, that he’s in pain, that he’s not ok. He goes to the middle of the room and gets on all fours on the floor and starts punching the floor, hard. Teresa gets up and tries to stop him. She knows Michael but also says “I have a brother like this, who’s sick like this, it's horrible.” She manages to get Michael off the floor and into a chair by the filing cabinets. He starts throwing his head back, slamming it into the cabinet. “Don’t do that!” Teresa is yelling. She looks around, “You don’t mind if he goes first do you, you can see he’s not ok. My brother is like this…” Teresa is telling the room not to be scared of him. He wouldn’t hurt anyone. Michael says this too, “I love you all, I’m not going to hurt you.” Teresa sits back down, “I can’t do this! I’m panicked enough as it is! I hate this fucking place.” A member of staff is there and brings Michael in to see the GP next door. When he comes back, he’s calmer and apologises for making a scene. I hear a client across the room say he must have been given something. (**Observation**) |
|  | Smoking | Pamela says, “I’m really sorry but I need to go out and have a cigarette. I can’t sit still for too long." (**Pamela, In-Depth Conversation**)  I walked in a fire exit door, past a three people smoking and chatting outside (two men, one woman). (**Observation**) |
| Health information | Reasoning behind care | One of the men in the group, a younger man, says that he’s on benzo maintenance (no methadone) but wants to be off in three weeks. He’s getting handed stacks of pills but doesn’t know why, “I’m trying to get off this stuff, I don’t want to be on these forever.” (**Observation**)  We talk about Ken’s healthcare. He’s trying to get a medical card. A member of staff is having to go through the process of attempting to ring 3GPs and have them say no before the HSE will work to help him find a GP. The GP tells Ken to come back on Wednesday morning to get his bloods done. “This is the third time they’ve told me to come back...” (**Ken, In-Depth Conversation**) |
|  | Addiction prevention | He says that if he could change something, it would be targeting people that aren’t too far gone on their addiction. He says you have to go into the community and find people who are just getting started and intervene sooner rather than later. He thinks that if someone had done that for him, he wouldn’t be in the clinic now. He says he had to refer himself. Timmy goes in to see the GP but says it to me again on his way out. "Make sure to tell the doctor that. That you have to get the young people. It’s really important." (**Timmy, In-Depth Conversation**) |
|  | Non-medicalised recovery options | Lorcan says the pain of facing your trauma is not as bad as the pain of addiction. He wishes people knew this. When GPs hear somebody say ‘I’m fed up. I can’t do this anymore’, he wishes they would point them towards stabilisation, residential treatment, 12-step. He says that a lot of people, even GPs, don’t know about the services available. They don’t know that there is a way to face the pain besides drugs and alcohol. In addiction, those seem like the only option. He wants people to know that it’s not. (**Lorcan, In-Depth Conversation**) |
| Opioid substitution therapy | Methadone overprescribing | Jack says that if he could change one thing, it would be how hard it is to get off methadone. He explains that now, a person starts on a lower dose until, say a few weeks in, they use street drugs. The doctors then raise the methadone dose. To him, this doesn’t make sense. If a person was having withdrawals so severe that they needed to take illicit drugs in addition to their methadone, that would have happened early on, not several weeks in. He doesn’t think raising the dose is the solution because it’s very hard to get off. (**Jack, In-Depth Conversation**) |
|  | Satisfaction with Opioid Substitution Therapy (OST) | I ask Lucy how her healthcare is going and if she feels she could be better supported anyway. She has little to say about her healthcare, at first. She says the clinic is good. She’s been on methadone for [nearly a decade] and hasn’t used during those. I ask her how she manages when there’s such easy access to drugs across the street from the clinic and around. She says something along the lines of “willpower.” (**Lucy, Casual Conversation**) |
| Mental healthcare | Dual diagnosis | Jack says that he went to a psychiatrist a day that he was suicidal and was sent back to the streets with two pills, one for the night and one for the morning. He took both immediately. The psychiatrist said that the suicidal thoughts were linked to his drug use and not his mental health. “That doesn’t make sense because I’ve been using drugs since I was a teenager but I’m only suicidal since my partner kicked me out.” (**Jack, In-Depth Conversation**)  “I can’t get psych medication until my addiction is sorted but the more I get my addiction sorted, the more it’s bringing up old emotional stuff that I need help dealing with.” (**Alannah, In-Depth Conversation**) |
|  | Unsatisfactory options | She says her rehab facility charged [nearly €100] a week and didn’t provide grief counselling, or any counselling. “I know grief”, she says. She is very upset about the loss of her brother. “Broke me heart, broken as it is.” (**Teresa, Casual Conversation**) |
|  | Criminal response | She tells me of one episode of psychosis so bad that she ended up covered in blood running in and out of cars. The police came but didn’t call the ambulance. “Why?” I ask. She laughs, “You think they’d come for me?! Having a psychosis, supposedly on drugs? That could take ages.” Instead, she was put into a jail cell until she was feeling well enough to leave. The police know her. (**Nora, In-Depth Conversation)** |
|  | Not ready to seek help | Towards the end of our conversation, I mention the clinic’s drop in psychotherapist and asks what she thinks about this. “Oh you know me, once you got me talking I just wouldn’t stop.” (**Alannah, In-Depth Conversation**) |
| **Theme III. Migrant Health** | | |
| **Sub-Theme** | **Codes** | **Sample Excerpts** |
|  | Legal status and rights | When Ken arrived in Ireland, he registered with International Protection Office only to find out that he wouldn’t receive housing until his application goes through, which can take months. He is homeless. The men with him are homeless. I ask where they manage to sleep. It seems like a mix between hostels, sleeping rough, or trying to crash with friends who have housing. Asylum seekers in City West (some of whom he knows) are not allowed to have any guests whatsoever. Ken is not allowed to work as an International Protection Applicant and yet he’s only receiving €38.80 a week to live on. This is to find housing, food, transport, everything. (**Ken, In-Depth Conversation**) |
|  | Work arrangements | Hannah tells me she is from East Africa. She’s been in Dublin for several months and is on her own. She is part of Direct Provision. Her family is split between her home country and other European countries. She works in a warehouse and is about to start a new job as a security guard as well. "When will you sleep?" I ask. She laughs, "On the bus!" (**Hannah, In-Depth Conversation**)  I speak casually for several minutes with an immigrant from South Africa (Man 14). He's working long hours. He goes on a long tangent about how Covid-19 is a hoax. He makes sure that I know he is different from the other people attending the clinic. He's never drank or used drugs a day in his life. (**Man 14, Casual Conversation**) |
|  | Language/survival skills | Gavin tells me about a man, a refugee, he met the other day. He said the refugee recognized him on the street and asked for help finding some cardboard and a marker to write up a sign saying that he [the refugee] was from Ukraine and homeless. Gavin found the supplies and started helping him ask people for money. He says the man was shocked when they made about €10 in five minutes. He explained that refugees coming in don’t have the street skills he’s had to use all his life. (**Gavin, Casual Conversation**) |
|  | Coping mechanisms | He was diagnosed as pre-diabetic in [his home country], was able to improve his glucose scores from walking. Now that he’s in Ireland, he finds he’s eating a lot of chocolate. (**Ken, In-Depth Conversation**) |
|  | Family ties | His wife and children, his family, are still [in his home country]. Ken says that it’s much harder than he expected in Ireland. In the back of his mind is his family back home and needing to try and get them out of his home country as well. (**Ken, In-Depth Conversation**) |
|  | Unresolved trauma | Ken has been in Ireland a month or two. He literally fled for his life… It took him months to get to Ireland. He says it was the hardest period of his life. When I asked how he came to be in Ireland he says that he had asked the ‘gatekeeper’ or whoever it was that decided his destination to be somewhere safe and where he could earn his bread and butter. He wanted to be in the safest place possible. (**Ken, In-Depth Conversation**) |
