# Supplementary material for: “Just a knife wound this week, nothing too painful”: An ethnographic exploration of how primary care patients experiencing homelessness view their own health and healthcare
Source: PLoS One. 2024 Jul 9;19(7):e0299761. doi: 10.1371/journal.pone.0299761 (PMC11232971; doi:10.1371/journal.pone.0299761)
Supplement: S1 File — (DOCX) [file pone.0299761.s003.docx]

**Health and Legal Terms**

**12-step**: A structured program designed to help individuals recover from addiction, compulsion, or other behavioural problems. Originally developed by Alcoholics Anonymous (AA), it includes 12 guiding principles for personal recovery and growth.

**Alcohol detoxification**: The process of eliminating alcohol from the body, typically involving medical supervision to manage withdrawal symptoms and ensure safety.

**Asylum seeker**: An individual who has fled their home country and is seeking international protection, but whose claim for refugee status has not yet been determined.

**Benzodiazepine**: A class of psychoactive drugs used to treat conditions such as anxiety, insomnia, and seizures. Common examples include Valium (diazepam) and Xanax (alprazolam).

**Direct Provision**: A system used in Ireland to provide accommodation, food, and essential services to asylum seekers while their applications are being processed.

**Dual diagnosis**: The condition of having both a mental health disorder and a substance use disorder simultaneously.

**GP (General Practitioner)**: A medical doctor who provides primary and continuing care to patients in a community. GPs treat a wide range of health issues and refer patients to specialists if needed.

**Guards**: Informal term for ‘An Garda Síochána’, the national police service of Ireland.

**Health Services Executive Ireland (HSE)**: Ireland’s national health service

**Housing Assistance Payment (HAP)**: A form of social housing support in Ireland that helps individuals and families pay their rent to private landlords.

**International Protection Office (IPO)**: The office responsible for processing applications for international protection (asylum) in Ireland.

**Key worker**: A professional, often in health or social care, who provides support and guidance to individuals with specific needs, such as those in recovery from addiction.

**Librium**: A brand name for chlordiazepoxide, a benzodiazepine used to treat anxiety and alcohol withdrawal symptoms.

**Low-threshold services**: Harm reduction-based health care centres targeted towards people who use substances. Low-threshold services make minimal demands on the patient and do attempt to control their intake of substances.

**Methadone**: A long-acting opioid used in opioid substitution therapy to treat opioid dependence and manage chronic pain.

**Opioid Substitution Therapy (OST)**: A treatment for opioid dependence that involves replacing an illegal opioid, such as heroin, with a longer acting but less euphoric opioid, such as methadone or buprenorphine (Suboxone).

**Psychosis**: A mental health condition characterized by a loss of contact with reality, leading to symptoms such as delusions, hallucinations, and impaired insight.

**Rehab**: Short for rehabilitation, this refers to the process of helping individuals recover from addiction or other physical or mental health conditions, often involving inpatient or outpatient programs.

**Sleeping rough**: The act of sleeping outside or in places not meant for habitation due to homelessness.

**Social welfare**: Government programs designed to provide financial assistance and support to individuals and families in need, including unemployment benefits, child benefits, and housing supports.

**Stabilisation**: In the context of addiction treatment, the process of achieving medical and psychological stability, often through medical interventions and support services.

**Suboxone**: A medication that combines buprenorphine and naloxone, used in opioid substitution therapy to treat opioid dependence.

**Irish Colloquial Terms**

**Cellotape**: The British and Irish term for adhesive tape, commonly used for sealing packages and wrapping presents.

**Credit Union**: A member-owned financial cooperative that provides savings, credit, and other financial services to its members. In Ireland, credit unions are known for their community focus and accessible services.

**Getting battered**: A colloquial term meaning to get hurt by being repeatedly hit.

**Kip**: Informal term for a messy or run-down place.
