# Supplementary material for: “Just a knife wound this week, nothing too painful”: An ethnographic exploration of how primary care patients experiencing homelessness view their own health and healthcare
Source: PLoS One. 2024 Jul 9;19(7):e0299761. doi: 10.1371/journal.pone.0299761 (PMC11232971; doi:10.1371/journal.pone.0299761)
Supplement: Attachment — Submitted filename: Response to Reviewers_R2.pdf [file pone.0299761.s005.pdf]

To Reviewer 1:

Thanks very much for the time you've taken to review this manuscript. Your thorough feedback has helped make it a much stronger piece. We see the reasoning behind each of your points and have addressed them where possible:

**Reviewer 1 Comments**	**Our Response**
However, there are still very little developed subtopics (results) such as the case of "criminal justice"; "navigating addiction". I consider that if there is no possibility of developing them in depth and they really have relevance and weight in the results as well as in the analysis, then leave them out. It is not a simple decision, but I suggest the authors to reflect on the necessity to present stronger evidence.	We can see your point and have expanded the sections on Criminal Justice and Navigating Addiction.<br><br>In relation to 'Navigating Addiction', participants did describe barriers and facilitators to recovery however these descriptions are complex and will be reported in a separate manuscript. In this piece, we keep in substance-use related elements that relate directly to the overarching theme of 'Self-Identified Priorities'. This choice was made to ensure conciseness and that the manuscript does not become too long.
The biggest detail I see in this version is that social stigma is mentioned in a superficial way and the evidence presented is not compelling to account for such a complex phenomenon that requires exploring in depth. There are not enough theoretical elements in the analysis to support the relevance of the issue of stigma, especially in these especially vulnerable groups. In this way, the proposals made for both research and public policies are not supported by the results. This is quite delicate, especially if we consider the great weight that stigma has in the response to needs and how it operates as an important barrier in accessing services and care. For this reason, in my previous review I commented that I find the analysis somewhat superficial, and this is explained by the large number of topics that are to be presented and that were explored, limiting the deepening of the understanding of the central themes.	We agree completely that stigma is a complex phenomenon that is relevant in the lives of these study participants. As mentioned above, we have expanded the sections on Criminal Justice and Navigating Addiction to ensure that participants' views on stigma are more fully described. Illustrative quotes are embedded throughout the manuscript to demonstrate how clients experience stigma when interacting with social and health services, the criminal justice system, and the general public.<br><br>There is a large body of existing research looking into the effect of stigma on the health of people experiencing homelessness and resulting implications for policy and practice[1]. Our aim with this study was to do something different to this by looking at the health and healthcare needs of people experiencing homelessness through their own lens. As such, we hope that the reviewer can agree that examining complex phenomenon of how stigma operates as a

[1] Reilly J, Ho I, Williamson A (2022) A systematic review of the effect of stigma on the health of people experiencing homelessness. Health & Social Care in the Community 30:2128–2141. https://doi.org/10.1111/hsc.13884

	barrier in accessing services and care beyond what participants themselves expressed falls outside the remit of this paper.<br><br>You mention that there are a large number of topics presented and explored. In fact, we believe this is a finding in itself. Participants we spoke, rather than thinking of the systemic inequities at play contributing to their circumstances, listed the wide array of challenges they were trying to navigate at any given moment. We believe that the analysis is reflective of the tone of data we collected: participants were matter of fact about those challenges and how they wanted them changed and spoke about their different priorities as distinct from one another. Thanks to the first round of reviews, the proposed conceptual framework now ties participants' views together to paint a more in-depth picture of participants' priority health and healthcare needs. Within this framework, stigma is intentionally placed at the same level as other barriers noted by participants. For those we spoke with, stigma experienced when interacting with various services and the general public was just one of many challenges faced when trying to navigate their day-to-day lives. Stigma was perceived to be no more impactful than other barriers such as violence, pain, mental health conditions, etc. As you point out, it's imperative that research looks into the complex drivers of health inequities amongst marginalised populations. However, results from this particular project are valuable in a different way by demonstrating how these barriers towards optimal health are actually experienced and prioritised by the population in question.<br><br>We believe that with the revisions we've made in this round of edits, the combined manuscript elements (thematic analysis results, illustrative quotes, proposed conceptual framework, supplemental files with relevant data excerpts) support the recommendations for policy and practice made.
Without a doubt, the fact of not having audio recorded the interviews also limits the quality of the data since the interviewer	Yes, we see your point. Not recording interviews was a methodological decision made based on the recommendations of our clinic partners and their

distracts his attention by taking notes and recording verbatim the speech of the informants. I think there is also greater elaboration in this sense not only in the limitations but in the section that was added on quality based on Guba-Lincoln's elaborations.	knowledge of how clients had reacted to recorded interviews in the past. As soon as the recording device came out, clients generally were far more unwilling to participate in a project or to open up. As well, both clinic partners, research supervisors and the lead researcher's institutional human research ethics committee felt that building trust through the use of more informal techniques was another means to ensure the female researcher's safety in a chaotic, inner-city environment. While this level of risk aversion may have had an impact on the recording of results, the safety of the researcher was the primary concern, with a secondary consideration being the enablement of an outsider becoming accepted in the facility to gather open, honest and frank disclosures from participants.<br><br>Upon your recommendation, we have been more transparent in our manuscript that this may have impacted the credibility of our data. To the section in question, we have added:<br><br>"Choosing not to audio record participants was, by design, intended to prevent inhibitions in open discussion by ensuring that participants felt neither controlled nor examined [22]. To increase the fidelity of her notes, the researcher reflected perceived critical points back to participants during and after interviews and wrote up field notes immediately after each visit using a standardised template. Readers should keep in mind nonetheless that the data presented come from the lead researcher's own notes and memory and are subject to some degree of recall bias [23]."<br><br>[22] Goedhart NS, Pittens CACM, Tončinić S, et al. Engaging citizens living in vulnerable circumstances in research: a narrative review using a systematic search. *Research Involvement and Engagement* 2021; 7: 59.<br>[23] Swain JM, Spire ZD. The Role of Informal Conversations in Generating Data, and the Ethical and Methodological Issues. *Forum Qualitative Sozialforschung / Forum: Qualitative Social Research*; 21. 28 January 2020. DOI: 10.17169/fqs-21.1.3344.
