# Supplementary material for: “Just a knife wound this week, nothing too painful”: An ethnographic exploration of how primary care patients experiencing homelessness view their own health and healthcare
Source: PLoS One. 2024 Jul 9;19(7):e0299761. doi: 10.1371/journal.pone.0299761 (PMC11232971; doi:10.1371/journal.pone.0299761)
Supplement: Attachment — Submitted filename: Response to Reviewer 3.pdf [file pone.0299761.s006.pdf]

| Reviewer 3 Comments | Our Response |
|---|---|
| **Title & Abstract** | |
| 'homeless clients' can be perceived as stigmatising and there is a push to use non-stigmatising, person-centred language like 'people experiencing homelessness' which should be considered in the title and throughout the paper. | Thank you for recognising the importance of using non-stigmatising language. We changed the title to:<br><br>***"Just a knife wound this week, nothing too painful"*****: an ethnographic exploration of how primary care patients experiencing homelessness view their own health and healthcare** |
| • 'people in homelessness' seems a bit of a strange term – people experiencing homelessness is more common. | Changed to 'people experiencing homelessness'. |
| • Usually an abstract would include some background/context to provide readers with information about why the study was needed/conducted, so I suggest a few sentences are added here to provide this detail. This would then contextualise the final sentence to explain why their needs should be prioritised. | This is a good point. To the abstract, we have added:<br><br>*Community health needs assessments (CHNA) involving qualitative techniques help tailor health services to the specific needs of the population groups for whom they are designed. In light of increasing health disparities amongst people experiencing homelessness (PEH) – and to ensure the integration of their voices into a larger CHNA – this study used an ethnographic approach…* |
| • It would be good to mention the health needs assessment methodology in the abstract too. | We now mention this methodology in the first two sentences of the abstract. |
| **Introduction** | |
| • You use the term 'people experiencing homelessness (PEH)' here so this should be used throughout including in title and abstract. | The language throughout the manuscript has been revised accordingly. |
| • Bracket missing on page 9, line 43. | Good catch! Revised. |
| **Methods** | |
| • Under participants and setting it would be helpful to have a bit more detail regarding the clinic in which the study was conducted – where located, how funded etc (especially for international readers unfamiliar with the Irish healthcare system). | We've added as much context as possible on this, however, as there are a limited number of these clinics in Ireland, we are limited in the amount of detail we can provide without running into privacy and confidentiality issues:<br><br>*Located in a densely populated urban area, the study site provides a walk-in general practitioner (GP) service on weekday mornings for patients who are experiencing homelessness or who are undocumented migrants. The clinic,* |

| | |
|---|---|
| | *funded by the charity sector and HSE, is part of a network of specialised, low-threshold homeless health services operating across Dublin, Galway, Cork, and Limerick. [Paragraph continues]* |
| • Purposive, critical case sampling – it would be good to have more detail, particularly in terms of what criteria were used in terms of purposive sampling and how this was achieved. | We have expanded the following paragraph in the **Participants and setting** section:<br><br>*Clinic medical or operational staff identified and introduced the researcher to potential 'critical cases' who were present in the waiting room and met the following criteria: (1) had experienced/were experiencing homelessness and potential co-occurring addiction, (2) had an established rapport with centre staff and were deemed to be of sound mental capacity, (3) expressed a voluntariness to chat with the researcher without coercion, and (4) spoke English. As the project progressed, clinic staff helped the researcher identify critical cases with yet-to-be-explored characteristics / experiences (e.g., clients a certain age/gender, clients sleeping rough, clients in early recovery).* |
| • Was the reflective notes template based on others' work or created for this study? It would be good to make this clear and add any references, if relevant. | That's a good point; we've added the relevant reference to the **Data collection** section. |
| • It would be good to discuss if informed consent was granted for the casual conversations and if not, why not. The same would be the case for observations. | In the **Ethical considerations** section, we clarify:<br><br>*In some instances, informed consent could not be obtained during casual conversations or participant observations due to the complex dynamics of the clinic space. In these instances, only non-identifying public data were recorded (e.g., statements made loud enough for the entire waiting room to hear) [31] and any collected data analysed and reported at the general level. Explicit consent was sought and obtained from all participants who are quoted in the manuscript.*<br><br>[31] Zahle J (2017) Privacy, Informed Consent, and Participant Observation. Perspectives on Science 25:465–487 |
| **Findings** | |

| | |
|---|---|
| • A note re. language on page 19, line 346 – alcoholism is an outdated and stigmatising term and should be avoided. I'd encourage you to change to 'problem alcohol use' or 'alcohol dependence'. | Revised to 'alcohol dependence'. |
| • Page 21, line 417 – suggest adding a definition to 'garda vetting' for non-Irish readers. | We've changed to more international language here: *"or failing to pass a criminal record check."* |
| • There are also several places where language used is not familiar with international readers (battered, kip) so a glossary might be useful. | This is a great suggestion. We've added a glossary of potentially unfamiliar terms as a Supplemental File. |
| • I would suggest the authors rethink the inclusion of theme 3 – it is somewhat different to the rest of the findings and is far less detailed – themes 1 and 2 are 7 and 4 pages each, respectively and theme 3 is less than 2 pages. It feels very different to the rest of the findings and doesn't really add as much depth. | This is interesting. The limited amount of data presented under the theme 'Migrant Health' is reflective of how participants who had recently migrated to Ireland were harder to reach/speak with than Irish clinic attendees. This was due to language barriers, and the fact that non-Irish clients were often new to the clinic and did not have the same relationship with staff (who were gatekeepers to study participation) as their Irish counterparts.<br><br>As part of the larger Community Health Needs Assessment we mention in the paper's introduction[1], we also spoke to health and addiction care professionals who – nearly unanimously – commented on the fact that there's a gap in what is understood in Ireland (and one would guess, across Europe) about the health needs of migrants and asylum seekers who are entering the country straight into homelessness due to the worsening housing crisis.<br><br>Despite being shorter than the other themes, the fact that Theme 3 provides some information on this issue and that the information provided reveals such stark health needs within this particular population, we feel merits its inclusion in the paper. We hope that the preliminary findings provided can serve as justification for researchers seeking to explore the issue in more depth. As a follow-up to this study, our team aims to target this particular cohort (which is rapidly expanding in Ireland as the number of asylum seekers steadily increases) of people experiencing homelessness to better understand their |

[1] Ingram C, MacNamara I, Buggy C, Perrotta C (2023) Priority healthcare needs amongst people experiencing homelessness in Dublin, Ireland: A qualitative evaluation of community expert experiences and opinions. PLOS ONE 18(12): e0290599. https://doi.org/10.1371/journal.pone.0290599

| | |
|---|---|
| | physical and mental healthcare needs as they navigate the asylum-seeking process in Ireland. |
