# Supplementary material for: “Just a knife wound this week, nothing too painful”: An ethnographic exploration of how primary care patients experiencing homelessness view their own health and healthcare
Source: PLoS One. 2024 Jul 9;19(7):e0299761. doi: 10.1371/journal.pone.0299761 (PMC11232971; doi:10.1371/journal.pone.0299761)
Supplement: Attachment — Submitted filename: Response to Reviewers.docx [file pone.0299761.s004.docx]

Dear Plos One Editors and Reviewers,

On behalf of our research team, we are very grateful for the thorough and helpful feedback provided regarding our manuscript, **“*Just a knife wound this week, nothing too painful”:* an ethnographic exploration of how homeless clients attending an urban primary care and addiction service view their own health and healthcare.** We have taken these suggestions very seriously and thoroughly reflected upon how to best incorporate them into the manuscript. Notable changes are as follows:

- To the methods section, we address the potential limitations to data collection mentioned by Reviewer 1 and provide a much greater level of detail on how data were systematised and analysed, and how criteria for rigour and trustworthiness were upheld.
- To the results session, we provide greater depth and clarity of analysis by including our coding framework, expanding illustrative excerpts within the text, and providing closure within and between sections.
- Finally, we tie thematic analysis results together with a Discussion section now dedicated to a more detailed interpretation/conceptualisation of results, strengthened by the addition and explanation of a Proposed Conceptual Model. The Policy Implications we have added– as suggested by Reviewer 2 – now stem directly, and we feel are justified, by participants’ own suggestions for how to improve their health and wellbeing.

We feel the manuscript has improved in terms of clarity and depth, and that conclusions made are thus now better supported by the findings reported. Homeless participants we spoke with expressed a desire to be better supported and heard. We hope that this manuscript will serve as one mechanism for ensuring that their views and voices reach and resonate with a wider audience. We are grateful to you, the editor and reviewers, for helping us do a better job of conveying their needs and experiences to that audience.

Thank you, on behalf of our research team.

| **Reviewer 1 Comments** | **Our Response** |
| --- | --- |
| Observation, what was observed, how the information was systematized? | To the Data Collection paragraph we have better described our field note template used to systematise the data:  *To increase fidelity, the researcher reflected back perceived critical points to the participant during interviews and wrote up formal fieldnotes immediately after each visit using a four-section template to standardise data collection. In the first section – Description of Activity – she recorded descriptive, open-ended notes on conversations held and observations made in the order of their occurrence. Conversations were delineated from observations using headings with the conversation type and participant’s name (later pseudonym). The latter three sections –Reflections, Emerging Questions/Analyses, Future Action -- provided space for analytic notes where, distinct from the descriptive data, the researcher began to interpret what was heard and observed.*  Under the sub-heading ‘Observations’ we have elaborated on the categories of things observed. We documented generally recommended categories of things to observe in line with Mack et al. (2005) p.20:  Mack N, Woodsong C, MacQueen K, et al. Qualitative Research Methods: A Data Collector’s Field Guide. North Carolina: Family Health International, USAID, 2005. |
| Due to the procedure in which the conversation was carried out, it does not differ much from the processes for conducting an interview (audiotaped) except for the issue of recording. What difference does it really make between recording or not? This poses many limitations in the quality of the data, how was it resolved?  Note taking can be as or even more intrusive than recording. Memory bias? How was it resolved? | We decided not to use audio recorders based on the recommendation of our clinic partners. Trust was built gradually between our research team and clinic attendees and the thinking was that coming straight into the project with a tape recorder could impede this trust building. This was especially true as many participants would go on to speak about sensitive topics like illicit drug use and assault. We made sure to ask if the participant was alright with notetaking as part of the informed consent process. All participants said yes to this with no hesitation. There were a few instances where a participant said something like “off the record” or “just between you and me” during an interview and no record of what was said next was kept.  To minimise memory bias, we reflected critical points back to the interviewee during the interview (*e.g., What I heard you say there was that…. Am I getting that right?*) or – if appropriate – took the time to write down a brief quote verbatim (*e.g., “Oh that’s a great point, give me one second as I write that down.”)* We made sure to write up field notes directly after each research visit so as not to lose information over time. Because many clinic attendees came regularly, we were able to follow up on several occasions about points of confusion/ideas we felt we hadn’t captured in full. To better describe these points, to the Data Collection section we have added:  *Data collection took place in the form of in-depth and casual conversations, and observations. The researcher chose not to record conversations during her visits as it could create concerns about confidentiality and privacy [26]. Instead, she jotted down raw records in a notebook (during observations and in-depth conversations or after casual conversations), a technique recommended for maintaining informality and building trust with vulnerable participants [27]. To increase fidelity, the researcher reflected back perceived critical points to the participant during interviews and wrote up formal fieldnotes immediately after each visit using a four-section template to standardise data collection…[…].* *When a point of uncertainty emerged during the writing up of interviews, it was marked as ‘needs follow-up’ and added to future actions. If the researcher crossed paths again in the clinic with the interviewee, she asked them to clarify the point in question or else used it to prompt discussion in other interviews.* |
| What strategies were followed to guarantee the quality of the data collected? | In addition to the clarifications made in the ‘Data Collection’ (described above), we have added a section called ‘Rigour and Trustworthiness’ to the methods which describes in more detail how Lincoln and Guba (1985)’s criteria for ensuring credibility, transferability, dependability, and confirmability were followed:  *Lincoln and Guba (1985)’s criteria for enhancing rigour based on credibility, transferability, dependability and confirmability were followed [33]. To ensure credibility, the lead researcher, who is formally trained in qualitative research techniques, spent a prolonged period at the study site (60 hours) and created the study protocol in collaboration with clinic partners with whom she also held regular debriefing sessions over the course of the project. The inclusion of in-depth conversations whereby purposively chosen participants were invited to speak freely about what was on their mind supported the construction of data representative of the authentic views of PEH. To ensure dependability, in addition to the description of methods presented here, the study protocol was pre-registered on Open Science Framework [34]. As part of a larger CHNA, confirmability was enhanced by triangulating study findings with previous literature published on similar cohorts in Ireland [2], and the perspectives of health and addiction professionals working with the same population during the study period [11]. The researcher’s field notes template included a section for personal reflections to encourage reflexivity. Finally, detailed field note excerpts are included in Supplemental Materials in addition to illustrative quotes embedded within the results to ensure that researchers can make judgements on the transferability of findings to other study populations [33].* |
| There were no references from the informants to other possible informants (snow ball sampling). Was there any other strategy to identify informants? Did you only trust the intuition and decision of the clinic staff? How did this decision influence the type of informants and the quality of the data? | Institutional ethical approval to conduct in-depth interviews was granted on the condition that clinic staff make introductions between the researcher and potential participants whom they deemed to be of sound mental capacity prior to the interview taking place. This was to protect individuals for whom talking about their health and other needs/experiences had the potential to be upsetting or distressing. It was also there as a protection mechanism for the researcher themselves. We recognize that this may have influenced the type of informants and have added to the Limitations section:  *Because participants who participated in in-depth conversations were referred to the research team by clinic staff with whom they had an established rapport, it is likely that the sample captures more favourable views of the primary care and addiction service than may have otherwise been the case.* |
| Taking notes regarding what a person says is already an exercise in synthesis and reflection that implies analysis. How was this issue worked on and resolved in the analysis carried out by two researchers? Was there any discussion about decision making regarding note taking? | You are exactly right. We have expanded our analysis section to describe how analysis began taking place in parallel to data collection:  *In line with the social constructivist process of iteration, analysis, critique, reanalysis, and synthesis [17], data analysis took place alongside data collection. Before each clinic visit, the researcher reviewed notes written in the ‘Emerging Questions/Analyses’ portion of her fieldnotes to determine where to guide conversations should clients seek to be prompted. This allowed for the progressive focusing of interviews and observations on issues that seemed particularly important or controversial to clients. Because the development of broad analytic categories that captured participants’ priority concerns and the mechanisms driving them emerged before data collection was complete, the researcher was able to run findings by other participants as a means of strengthening credibility. The researcher also regularly presented clients’ views to the wider research team and clinic partners to gain clarity on whether expressed concerns matched the realities of the services provided….*  *Once data collection was complete, field note data (80 pages, 40,000 words) were coded and analysed according to recommended stages of trustworthy, inductive thematic analysis [35–37]. Two researchers (CI,IM) familiarized themselves with the data by re-reading and annotating the descriptive sections of the field notes. The researchers came together to discuss their initial analytical notes, thoughts, and impressions. By design, CI waited to share her sense of emerging broad analytic categories until her colleague had formed her own initial impressions of the data…* |
| The presentation of the analytical process in the form of bullets seems to oversimplify it. I suggest a rich description of the process and the way decisions were made. | We have thoroughly revised our Analysis section to provide a richer description of the process and the way decisions were made. Please see this section in the attached Manuscript. |
| Was the same data treatment given to the conversations as to the observation? Were the codes the same for conversations as for observation? How was this achieved when it is difficult to account for experiences through observation? How did the observation allow us to know the housing status, living with relatives and other characteristics? Are they the same interview participants? | Data from conversations were delineated from observational data in the descriptive portion of the field notes. The two analysts coded these notes as one piece, but it was possible to compare the codes applied to observations and conversations. Quickly, we observed substantial overlap of the codes regardless of data type, which influenced our decision to keep all data types together as one data set. We now better explain this in the second paragraph of the Analysis section. In regard to your point that it is difficult to account for experiences through observation, we would argue that observation very often gave us valuable insight into participants’ experiences. As one example, the study site allowed us to observe how clients experienced pain as they waited, oftentimes doubled over, or wincing, or holding their wound. We considered these observations to depict truth of experience to the same extent as a participant describing their pain in conversation.  Your curiosity about how we observed data on housing status etc. is understandable! We were surprised by how much we could learn just by sitting in the waiting room. Clients would often say to a friend where they were staying or what they had taken that week; comments that were loud enough for the whole room to hear and allowed us to understand the observed participant’s context. This wasn’t true for everyone we observed, which is why there are missing data in Table 2. Participants in the ‘observed’ category are not the same as participants with whom we held conversations. If we initially observed someone who later was referred for an in-depth conversation, we classified them under ‘in-depth conversation.’  To the observations section, we have added:  *Because clients chatted openly amongst themselves or on the phone while waiting, it was possible to observe and record information on housing status and nationality. Information on substance use, when not overheard, was observed as clients handed over urine samples, picked up methadone scripts, were visibly intoxicated, or experienced withdrawal while waiting.* |
| How is figure 1 constructed? The lines have no direction. Analysis of the figure is missing in the text. | Figure 1 is no longer in the manuscript as we now provide a more detailed analysis and resulting conceptual model. We will explain these changes in detail below. |
| The identification of needs lacks a qualitative analysis. Was it quantified according to the number of times it is mentioned? How those needs were defined. How is a count of mentions justified according to the analytical framework proposed by the study?  Much of the analysis in the following sections is supported by the number of informants who mention the topic. They even refer to the number of people who say it or refer to the majority. Is the relevance of the topics only due to frequency or how was the decision | Thanks very much for pointing this out. Despite having completed a thorough thematic analysis, we (for some reason that is now confusing to us!) reported results in a way that did not make this clear. We have attempted to remove all mentions of count from the thematic analysis results, as each topic was chosen as relevant not based on the number of times it was mentioned, but *that* it was mentioned and expressed as being important to a/multiple participants.  We have thoroughly revised our analysis and results section to reflect this. Namely, we provide a richer description of our analysis process; have added the codebook as Figure 1 which depicts emergent themes, sub-themes, and codes; and provide a description of each theme at the start of the Themes 1, 2, and 3 sections. |
| I do not find sufficient elements to assess the relevance of the topics beyond the number of people who mention them. At least it is not present in the document. In that sense, how did you choose the interview excerpts that are presented?  There is an important difference in the development and inclusion of testimonies between topics and in general the analysis remains at a descriptive level. | We have added additional interview excerpts to the results section to aid the reader in better assessing the relevance of the topics. In doing so, we have attempted to find a balance between conciseness and ensuring that participants’ views and voices are sufficiently understood as they relate to the themes. We have added the following paragraph to our results section to explain how quotes were selected:  *In the following sections, we focus on three main themes emerging from our analysis: self-identified priorities, satisfaction with services, and migrant health. Figure 1 summarises the broad themes, sub-themes, and codes. Illustrative data extracts from the lead researcher’s field notes are provided throughout the text for each sub-theme and labelled by participant pseudonym. Embedded and longer quotations were selected with the aim of invigorating the text and illustrating diverse participants’ complex experiences. For conciseness, we reserved the use of longer quotations for findings not often referred to in the literature [37]. The complete coding framework, which provides more granular illustrative examples for each code, is available in Supplemental Materials.*  Readers that wish to see more interview excerpts underpinning each code/sub-theme/theme can refer to the detailed Codebook that is now presented as Supplemental Material. |
| There is no closure in the different sections of the results. | We have added a brief closing sentence/paragraph to each section of the results that summarises the main findings within that section. |
| Where do the subtopics come from? How do you get to them when only three were presented as relevant without mentioning the important list of their topics? | Figure 1 depicts how overarching themes and sub-themes emerged directly from codes applicable to the dataset. |
| This work lacks the depth sought through an ethnographic study. It lacks a conceptual framework that allows relating the findings of the study no matter how much of an ethnographic approach it has sought to have. The fact of posing a question and a problem suggests having a frame of reference. Nor are there elements in the analysis that suggest or allow us to identify the framework proposed by social constructionism. | This is a really, really helpful piece of feedback that we are grateful to have received.  Since receiving this peer review, we have worked as a team to revisit findings and interpret/conceptualise them in greater depth. This is reflected in our addition of a Proposed Conceptual Model (Figure 2) to the manuscript, with an accompanying analysis in the text. These additions seek to better answer the question of, not only ‘What did clients say?’, but ‘Why does what they say matter?’ We ask that Reviewer 1 refer to this section directly in the manuscript.  The framework of social constructivism is incorporated into this analysis in the sense that we used an inductive, data-driven approach where themes were derived and conceptualised from the data itself without the influence of pre-existing theories ([Braun & Clarke, 2006](https://journals-sagepub-com.ucd.idm.oclc.org/doi/10.1177/16094069231205789#bibr5-16094069231205789), [Naeem et al., 2023](https://doi-org.ucd.idm.oclc.org/10.1177/16094069231205789)). |
| It would be very interesting to know why they refer to informants as “clients.” | We refer to participants as clients based on the terminology used by clinic partners and across the Irish homeless services sector. |
| The discussion is little supported by the results presented given the superficiality of the analysis. The same with the conclusions and recommendations they make. | We have shortened the Discussion section to focus only on implications that stem directly from participant’s own suggestions for what could contribute to their safety, health, and well-being. We hope that we have now provided sufficient depth of analysis and reporting to demonstrate how these implications reflect participants’ self-identified needs and wishes. |
| **Reviewer 2 Comments** | **Our Response** |
| I would like to thank the authors for giving me the opportunity to review this high-quality work. The article is interesting and takes a qualitative approach to a subject relating to health services and addiction. The design is particularly well suited to exploring the user's point of view. I recommend this article for publication in Plos One with some minor comments.  I have a few comments to make to improve the work. I think that the results in terms of health policy implications could be highlighted more in the discussion. I think that if these results are interesting, how can they be articulated with the organization of care from a practical point of view? How can these results enlighten health policy regulators? | Thank you, Reviewer 2, for this feedback. We have revised our Discussion section to include a section on Policy Implications that reflect participants’ own self-identified suggestions for how to improve their safety, health, and well-being. |
